# Saturation of electrical resistivity of solid iron at Earth’s core conditions

**DOI:** 10.1186/s40064-016-1829-x

**Published:** 2016-03-01

**Authors:** Monica Pozzo, Dario Alfè

**Affiliations:** Department of Earth Sciences, and Thomas Young Centre@UCL, University College London, Gower Street, London, WC1E 6BT United Kingdom; Department of Physics and Astronomy, and London Centre for Nanotechnology, University College London, Gower Street, London, WC1E 6BT United Kingdom

**Keywords:** Earth’s core, Ab initio calculations, DFT, Electrical resistivity, Saturation

## Abstract

We report on the temperature dependence of the electrical resistivity of solid iron at high pressure, up to and including conditions likely to be found at the centre of the Earth. We have extended some of the calculations of the resistivities of pure solid iron we recently performed at Earth’s core conditions (Pozzo et al. in Earth Planet Sci Lett 393:159–164, 2014) to lower temperature. We show that at low temperature the resistivity increases linearly with temperature, and saturates at high temperature. This saturation effect is well known as the Mott-Ioffe-Regel limit in metals, but has been largely ignored to estimate the resistivity of iron at Earth’s core conditions. Recent experiments (Gomi et al. in Phys Earth Planet Int 224:88–103, 2013) coupled new high pressure data and saturation to predict the resitivity of iron and iron alloys at Earth’s core conditions, and reported values up to three times lower than previous estimates, confirming recent first principles calculations (de Koker et al. in Proc Natl Acad Sci 109:4070–4073, 2012; Pozzo et al. in Nature 485:355–358, 2012, Phys Rev B 87:014110-10, 2013, Earth Planet Sci Lett 393:159–164, 2014; Davies et al. in Nat Geosci 8:678–685, 2015). The present results support the saturation effect idea.

## Background

The thermal and electrical conductivities of iron at Earth’s core conditions (pressures between 135 and 364 GPa and temperatures between 4000 and 6000 K) are the two key parameters needed to determine the fundamental time scales for diffusion of heat and magnetic field in the core of our planet. Traditionally, these two parameters have been obtained by extrapolations from near ambient conditions (Stacey and Anderson 2001; Stacey and Loper 2007) but recent calculations (Pozzo et al. 2012; de Koker et al. 2012; Pozzo et al. 2013, 2014; Davies et al. 2015) and experiments (Gomi et al. 2013; Ohta et al. 2014, 2015) have shown that those early estimates provided conductivity values that were too small by a factor of 2 or 3. A very recent study carried out on the zero temperature perfect crystal at ICB pressure suggested that a new effect, due to electron-electron scattering, would reduce the conductivity back to the old values (Zhang et al. 2015). It is not clear if a calculation on a perfect crystal has relevance to the liquid Earth’s core (or even to the high temperature solid), and so for the purpose of this study we will not discuss these results further.

The main reason for the much higher conductivities suggested by the recent experiments of Gomi et al. (2013), later confirmed by direct measurements by Ohta et al. (2014, 2015), was the inclusion of saturation effects in the extrapolation of the conductivities at high temperature. This effect had not been taken into account before, leading to the much lower estimates of Stacey and Anderson (2001) (and subsequently of Stacey and Loper 2007). The saturation effect is well known in metallurgy, but Gomi et al. (2013) were the first to include it for iron at Earth’s core conditions. It is based on the idea that in the semiclassical approximation the resistivity of a metal is inversely proportional to the mean free path of the electrons. Since the mean free path is inversely proportional to temperature, then the resistivity is increasing linearly with temperature (the Bloch-Gruneisen approximation). However, if the electrons are only scattered by phonons and impurities in the system, the mean free path can only decrease up to the interatomic distance, the so called Ioffe-Regel condition (Ioffe and Regel 1960). At this point the resistivity saturates and stops increasing with temperature. In principle electron-electron scattering is still possible at shorter distances, and quantum effect could mean that the Ioffe-Regel condition can be violated, and indeed it is violated in some strongly correlated materials like high-Tc cuprates and alkali-doped fullerides, but for simple metals it is expected to hold quite well (see e.g. Gunnarsson et al. 2003 for a review). Resistivity saturation effects have just been reported in Gd and Fe from first principles calculations at ambient pressure by Glasbrenner et al. (2014). Recent theoretical calculations predicted resistivities of iron and iron alloys at Earth’s core conditions (de Koker et al. 2012; Pozzo et al. 2012, 2013, 2014; Davies et al. 2015) that were later found to be very close to those estimated by Gomi et al. (2013), supporting the idea of high temperature saturation. Direct measurements by Ohta et al. (2014, 2015) are also in good agreement with these estimates. These results were further supported by a recent stochastic model for fluctuations in the Earth’s dipole field (Buffett et al. 2014; Buffett and Matsui 2015).

Here we have extended some of the calculations of the resistivities of pure solid iron performed at Earth’s core conditions (Pozzo et al. 2014) to lower temperatures, to study the temperature behaviour of the resistivity. We found clear evidence of the saturation effect.

## Techniques

The DFT technical details used in this work are similar to those used in recent papers (Alfè et al. 2012; Pozzo et al. 2012, 2013, 2014). First principles simulations were performed using the vasp code (Kresse and Furthmuller 1996), with the projector augmented wave (PAW) method (Blöchl 1994; Kresse and Joubert 1999) and the Perdew-Wang (Wang and Perdew 1991; Perdew et al. 1992) functional (PW91). We used an iron PAW potential with the $4s^13d^7$ valence configuration, with a core radius of 1.16 Å. Single particle orbitals were expanded in plane-waves with a cutoff of 293 eV. Electronic levels were occupied according to Fermi-Dirac statistics. The details of the electronic temperature will be discussed in the “Results” section. An efficient extrapolation of the charge density was used to speed up the ab initio molecular dynamics simulations (Alfè 1999), which were performed on super cells of hexagonal-close-packed (hcp) iron including 150, 288 and 490 atoms, and sampling the Brillouin zone (BZ) with the $\Gamma$ point only. The effect of simulation cell size on the resistivities will be discussed in the “Results” section. The temperature was controlled with an Andersen thermostat (Andersen 1980) and the time step was set to 1 fs. We ran simulations for typically 8–13 ps, from which we discarded the first ps to allow for equilibration. We then extracted 40 configurations separated in time by 0.25 ps, which guarantees that they are statistically uncorrelated, and calculated the resistivities on these ionic configurations using the Kubo-Greenwood formula, as implemented in vasp by Desjarlais et al. (2002). Convergence of the resistivity with respect to BZ sampling will be discussed in the “Results” section.

## Results

We calculated the resistivity of hcp iron at the two volumes 6.84 and 8.95 Å^3^, corresponding to a pressure of 365 GPa at 6350 K and 97 GPa at 4350 K, respectively. For the low pressure point the calculations have been repeated at the temperatures 3350, 2350, 1850, 1350, 850 K and for the high pressure point at the temperatures 5350, 4350, 3350, 2350, 1850, 1350 and 850 K. The effect of simulation cell size on the resistivities has been tested using 150 and 288 atoms for the high pressure point, and using 288 and 490 atoms for the low pressure point. The effect of **k**-points sampling has been tested for the high pressure point only by calculating the resistivity values using 2 and 6 **k**-points.

**Fig. 1:**
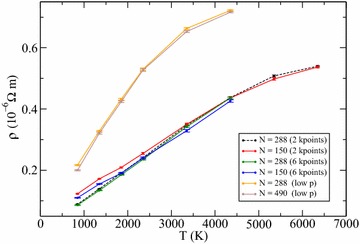
Electrical resistivity (*ρ*) of pure solid iron as a function of temperature (T). Results for the high pressure points (see also Table 1) were obtained using 150 and 288 atoms systems, using 2 and 6 **k**-points. Results for the low pressure points (see also Table 2) were obtained using 288 and 490 atoms systems with 2 **k**-points

Results are displayed in Fig. 1, with converged values listed in Tables 1 and 2. For the high pressure points, modest size effects appear at the lowest temperatures, as expected because the mean free path is longer, but at high temperature results converge quite quickly even with the smallest simulation cells containing only 150 atoms. Convergence with respect to **k**-points is also quick, showing that 2 **k**-points are enough for simulation cells of 288 atoms over the whole range of temperature studied at both pressures.

We observe that the resistivity increases linearly with temperature, initially, and then clearly shows the onset of a saturation behaviour for both the low p points (about 60–100 GPa) and the high p points (about 300–365 GPa) investigated.

**Table 1:** Temperature (T), pressure (P) and electrical resistivity (*ρ*) of pure solid iron

T (K)	P (GPa)	$\rho$ ($10^{-6} \,\Omega\hbox{m}$)
6350	365	0.540 (2)
5350	351	0.508 (3)
4350	338	0.437 (3)
3350	326	0.347 (3)
2350	315	0.241 (2)
1850	310	0.191 (2)
1350	306	0.139 (1)
850	301	0.089 (1)

Results are those obtained for systems of 288 atoms, with V = 6.84 Å$^3$  and 2 **k**-points. The reported temperature is the same for both ions and electrons

**Table 2:** Same as in Table 1, but for systems of 490 atoms, with V = 5.85 Å$^3$  and 2 **k**-point for low pressure values

T (K)	P (GPa)	$\rho$ ($10^{-6} \,\Omega\hbox{m}$)
4350	97	0.716 (2)
3350	85	0.653 (3)
2350	73	0.527 (4)
1850	68	0.424 (3)
1350	63	0.321 (3)
850	59	0.200 (2)

We now come to discuss how these results compare with previous literature studies. As mentioned in the introduction, the conventional values of the resistivity of iron at high pressure and temperature are, for example, those reported by Stacey and Anderson (2001), who estimated the resistivity of pure iron at Earth’s core-mantle boundary conditions (CMB; 135 GPa and 3750 K) and inner-core boundary conditions (ICB; 330 GPa and 4971 K) by taking low pressure/temperature values and extrapolating to core values, assuming constant resistivity on the melting curve. They obtained $\rho =1.22$ and $\rho = 1.12 \times 10^{-6} \,\Omega\hbox{m}$ at the CMB and the ICB, respectively. These are roughly 2 times higher than the values reported here and in previous papers (de Koker et al. 2012; Pozzo et al. 2012). Shock wave experiments performed by Bi et al. (2002) reported $\rho =0.7$ and $\rho =1.3 \times 10^{-6} \,\Omega\hbox{m}$ at 100 and 208 GPa, with estimated temperatures on the Hugoniot of 2010 and 5220 K respectively. Older shock wave experiments by Keeler and Royce (1971) reported $\rho = 0.67 \times 10^{-6} \,\Omega\hbox{m}$ at 140 GPa. A direct comparison with these shock wave experiments is not possible because the conditions of the Hugoniot are different from those reported in the present work, but our results are more compatible with the Keeler and Royce (1971); Sha and Cohen 2011) performed first-principles calculations of the electrical resistivity of a perfect hexagonal-close-packed (hcp) crystal of iron using the low order variational approximation. High temperature values were obtained using the Bloch–Gruneisen formula, which assumes linear increase of the resistivity with temperature. At Earth’s inner core conditions, they reported resistivity values between 0.7 and 1.5 $\times 10^{-6} \,\Omega\hbox{m},$ in the p and T range of 330–360 GPa and 5000–7000 K. It is worth mentioning that within the low order variational approximation, the resistivity is inversely proportional to the electronic density of states at the Fermi energy (DOS$_{E_F}$). For iron at Earth’s core temperatures the DOS$_{E_F}$ strongly depends on thermal disorder, and it is approximately 40% lower if computed on the perfect crystal, leading to a corresponding overestimate of the resistivity. Gomi et al. (2013) performed DAC high pressure experiments on Fe and Fe alloys. By including the saturation effect in the extrapolation of the measured electrical resistivity to high temperatures, they found resistivity values of about 0.4, 0.5, 0.5 and 0.4 $\times 10^{-6} \,\Omega\hbox{m}$ at 101 GPa/2010 K, 135 GPa/3750 K (CMB), 208 GPa/5220 K and 330 GPa/ 4971 K (ICB) respectively. These results have been recently confirmed by Ohta et al. (2014, 2015), who performed electrical and thermal conductivity measurements on iron up to Mbar pressures and temperatures up to 4500 K using a laser-heated diamond anvil cell, and explicitly observed the saturation effect. These new experimental results suggest smaller resistivities than conventional estimates, and in agreement with the values reported in this work.

## Conclusions

We have extended our recent calculations of the resistivity of pure solid iron performed at Earth’s core conditions (Pozzo et al. 2014) to lower temperatures to investigate the temperature behaviour of the resistivity. Our results show that the resistivity begins to saturate at high temperatures, and support the analysis included in recent experimental work (Gomi et al. 2013; Ohta et al. 2014, 2015).
